# Hierarchical Compliance Control of a Soft Ankle Rehabilitation Robot Actuated by Pneumatic Muscles

**DOI:** 10.3389/fnbot.2017.00064

**Published:** 2017-12-04

**Authors:** Quan Liu, Aiming Liu, Wei Meng, Qingsong Ai, Sheng Q. Xie

**Affiliations:** ^1^School of Information Engineering, Wuhan University of Technology, Wuhan, China; ^2^Key Lab of Fiber Optic Sensing Technology and Information Processing, Wuhan University of Technology, Wuhan, China; ^3^Department of Mechanical Engineering, University of Auckland, Auckland, New Zealand; ^4^School of Electrical and Electronic Engineering, University of Leeds, Leeds, United Kingdom; ^5^School of Mechanical Engineering, University of Leeds, Leeds, United Kingdom

**Keywords:** soft rehabilitation robot, pneumatic muscles, compliance adaptation, admittance control, patient-cooperative training

## Abstract

Traditional compliance control of a rehabilitation robot is implemented in task space by using impedance or admittance control algorithms. The soft robot actuated by pneumatic muscle actuators (PMAs) is becoming prominent for patients as it enables the compliance being adjusted in each active link, which, however, has not been reported in the literature. This paper proposes a new compliance control method of a soft ankle rehabilitation robot that is driven by four PMAs configured in parallel to enable three degrees of freedom movement of the ankle joint. A new hierarchical compliance control structure, including a low-level compliance adjustment controller in joint space and a high-level admittance controller in task space, is designed. An adaptive compliance control paradigm is further developed by taking into account patient’s active contribution and movement ability during a previous period of time, in order to provide robot assistance only when it is necessarily required. Experiments on healthy and impaired human subjects were conducted to verify the adaptive hierarchical compliance control scheme. The results show that the robot hierarchical compliance can be online adjusted according to the participant’s assessment. The robot reduces its assistance output when participants contribute more and *vice versa*, thus providing a potentially feasible solution to the patient-in-loop cooperative training strategy.

## Introduction

Stroke has been a seriously big healthy problem worldwide, which is believed to be the leading cause of death and disability, especially among elderly population and in low- and middle-income countries (Johnson et al., 2016). China has an increasingly aged and stroke population, the percentage of Chinese above 60 is expected to reach 39% of the population by 2050 (United Nations, 2016). As these demographics increase, there are increased demands for rehabilitation but decreased availability of people who can deliver rehabilitation. It is in desperate need of new technologies for adequate and affordable care for stroke patients. Robot-assisted solutions have the potential to alleviate the burden of post-stroke rehabilitation (Meng et al., 2015). It is well-recognized that inherent compliance of robot will help realize the safe human–robot interaction in rehabilitation. Combining traditional electromechanical drivers with elastic springs or directly using actuators with inherent compliance such as the pneumatic muscles is a potential way to ensure the compliance in human–robot environments (Hussain et al., 2013a; Wilkening et al., 2015), while the compliance offers a promising means to improve a robot’s soft dynamics and inherent safety.

A majority of existing Ankle Rehabilitation roBOTs (ARBOTs) are driven by rigid and stiff actuators such as electric motors. A typical ARBOT with three translational and three rotational degrees of freedom (DoFs), Rutgers Ankle, is driven by electric cylinders (Deutsch et al., 2001). Anklebot (Interactive Motion Technologies, Inc., USA) is a newly developed ARBOT driven by two linear actuators mounted in parallel and can actuate two DOFs, including ankle dorsi/plantar flexion and inversion/eversion (IE) (Ibarra et al., 2014). Saglia et al. also presented a high-performance ARBOT with two rotational DOFs driven by DC motors (Saglia et al., 2013). These rigid actuators along with passive training scheme may produce undesired large torques or forces in response to uncertain movements due to patient’s spasms (Hussain et al., 2013b) and, in this situation, the patient may feel confined, uncomfortable, or even painful. Choi and Lee (2010) indicated that the essential limitations to the human–robot interaction safety have been encountered owing to the lack of compliant actuators in robot design. For rigid rehabilitation robots, an impedance controller with proper stiffness and damping must be adopted to make the robot possess certain compliant features. The ARBOT employed an admittance control scheme to perform the patient-active exercise (Saglia et al., 2013). Lokomat (Duschau-Wicke et al., 2010a) and LOPES (Veneman et al., 2007) both applied impedance controller to achieve certain compliance of the robot when interacting with human users. Jutinico et al. (2017) developed an impedance controller for an ARBOT driven by series elastic actuators (SEAs). By adjusting the impedance of the robot, its behavior can be adjusted from very stiff to very compliant. However, this kind of compliance is “virtual,” as it only reflects the robot softness when the end effector interacts with the environment, not the compliance of the robot itself. Such a “virtual” compliance cannot guarantee sufficient softness of human–robot interaction due to the absence of actuator compliance and possess a very limit compliance in joint. Even in some situations the impedance model can provide certain virtual compliance in task space, this attempt may add an extra layer of control complexity to the robot and probably lead to rigid interaction again when several control actions unexpectedly fail (Sugar et al., 2007).

To tackle the problems existing in rigid actuators, researchers have tried to investigate pneumatic muscle actuators (PMAs) (Tsagarakis and Caldwell, 2003; Tondu et al., 2005; Beyl et al., 2009; Hussain et al., 2012). PMA has superior features in terms of lightweight and high power/volume ratios, which can offer the advantage of intrinsic softness to make the joint compliance possible, and thus is a promising intervention and has great potentials in rehabilitation robotics field. Hussain et al. (2013c) designed a lightweight robotic orthosis that is driven by PMAs and developed an adaptive impedance control strategy in task space to provide interactive robotic gait training. Park et al. (2014) also developed an active soft ankle orthotic device using soft actuators. A knee-ankle-foot orthosis that is bio-inspired driven by PMAs and controlled using patient’s lower limb myoelectric (EMG) signal was proposed by Sawicki and Ferris (2009). RUPERT is another four actuated DOF upper extremity exoskeleton driven by compliant PMAs (Sugar et al., 2007). Recently, Zhang and colleagues from Auckland research group developed a compliant ankle rehabilitation robot (CARR) actuated by four Festo pneumatic muscles (Meng et al., 2017a; Zhang et al., 2017a). These systems usually applied impedance-based controllers to achieve the desired movements (Lerner et al., 2017), however, the potential of PMA in design of fully compliant robot and how to realized complete compliance control have not been deeply explored. Similar to the control on traditional rigid actuators, the current compliance strategy on PMAs is still achieved by the use of impedance control in task space (Jamwal et al., 2016; Hussain et al., 2017; Zhang et al., 2017b). Actually, pneumatic muscles allow controlling the compliance behavior of each muscle separately, which is the “real” compliance since each muscle’s stiffness property can be regulated by pressure. Such intrinsic compliance of these actuators makes them ideal candidate for providing “real” compliant actuation for human–robot interaction applications (Kong et al., 2009). This is very important, especially in case of patient’s neural/physical rehabilitation, to provide the injured joint with required softness and compliance. The lightweight and compliance nature of pneumatic muscles has the potential to facilitate a more natural interaction with the patient (Awad et al., 2017).

In general, the current researches on compliance control are mainly focused on the robot impedance behavior when interact with external environment, which does not take the actuator compliance into account (Blaya and Herr, 2004; Duschau-Wicke et al., 2010b; Fleerkotte et al., 2014; Renquan et al., 2014; Jamwal et al., 2016). For soft robot-assisted neurological rehabilitation, not only the impedance in task space but also the compliance of the actuators and the robot itself is of crucial importance to ensure safety. To provide required compliant environment for specific patients, the robot’s inherent compliance and its impedance/admittance behavior should be separately controlled. With the objective to combine these two essential compliance properties, in this paper a new hierarchical control structure using nominal pressure adjustment and admittance controller is presented as the first specific solution to provide compliance in both task and joint spaces. The joint level controller attempts to adjust the actuator compliance *via* nominal pressure regulation based on the estimate of human active torque, and the task level one searches for an optimal balance between patient deviation freedom and the movement errors. For both strategies, the robot assistance level will be adapted according to the patient’s performance, effort, and ability. An adaptive compliance control scheme is applied to the soft ARBOT. The adaptive assistive controller learns online to adjust the robot compliance behavior by taking into account the patient’s active effort and movement ability while encouraging the patient’s voluntary effort to a maximum extent. Practical experimental tests with healthy subjects verify the feasibility and effectiveness of the proposed control scheme in helping patients to complete the training tasks with maximum voluntary active contribution and optimal assistance from the pneumatic robot.

The contributions of this work include the development and evaluation of a two-level compliance control scheme for an intrinsically compliant robot actuated by PMAs for multiple DOF ankle rehabilitation. The proposed control scheme allows the robot joint compliance to be controlled independently of the end effector compliance and movement. The joint compliance control provides enhanced safety for the patient–robot system without loss of assistance performance in task training. According to the authors’ best knowledge, compliance control of a soft rehabilitation robot in both joint and task spaces has not been reported in the literature. Based on this work, we can further explore the patient-cooperative control strategies for robot actuated by soft actuators.

The rest of this paper is organized as follows: Section “Ankle Rehabilitation Robot” demonstrates and models the PMAs-driven soft ARBOT. In Section “Hierarchical Compliance Controller,” the proposed hierarchical compliance controller on the basis of joint nominal pressure adjustment and task space adaptive admittance is presented. Experiments of ARBOTs with human participants are presented in Section “Hierarchical Compliance Controller,” followed by the results discussion, and the conclusion is drawn in Section “Experiments and Results Discussion.”

## Ankle Rehabilitation Robot

### Robotic Configurations

**Figure 1:**
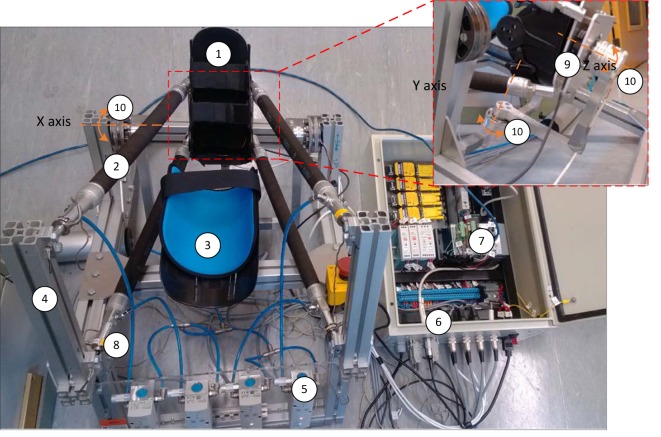
Prototype of the soft ankle rehabilitation robot (ARBOT): (1) end effector, (2) pneumatic muscle actuator (PMAs), (3) leg holder, (4) fixed platform, (5) proportional pressure regulator, (6) control box, (7) embedded controller, (8) single-axis load cell, (9) six-axis force/torque sensor, and (10) rotary encoders.

The current prototype of the soft ARBOT developed by the University of Auckland is shown in Figure 1 (Zhang, 2016). This robot is powered by four PMAs equipped in parallel to realize three DOFs for ankle rotational movement, that is, dorsiflexion/plantarflexion (DP), inversion/eversion (IE), and adduction/abduction (AA), respectively. FESTO™ pneumatic muscles with model FESTO DMSP-20-400N are adopted to guarantee the intrinsic compliance and force generation ability of the robot during operation. Four high dynamic pneumatic regulators (FESTO VPPM-6L-L-1-G18-0L6H) are used to regulate the pressure inside each muscle. The end-effector is a three-link serial structure with magnetic encoder (AMS AS5048A) installed in each joint to measure the angular positions in Euler X (DP), Y(IE), and Z (AA) axes of the robot end-effector. Joint encoders are used to measure the ankle rotary displacements which can be used to calculate the lengths of PMAs by inverse kinematics.

In this study, we will concern the actuated DOFs in ankle DP and IE movements and the AA DOF will be kept passively free during operation. The maximum ranges of motion for ankle DP and IE are 38/38 degrees and 24/24 degrees, respectively. The robot frame supporting the weight of the robot and human shank and ankle is adjustable to suit different patients. PMAs are applied for providing actuation to the robot, with the rest length of 400 mm and a maximum stroke of 100 mm. Each PMA can provide a peak of 1,000 N pulling force and the produced joint torque can reach up to 30 Nm, which is sufficient for the proposed application. A force sensor (Futek LCM 300) is placed in series with each PMA to test the pulling force and a six-axis load cell (SRI M3715C) is mounted between the human and robot to measure the interaction torque. All the hardware is controlled by using an NI Compact RIO-9022 system and the control system run on a host PC using LabVIEW. Several safety considerations have also been embedded in the robotic hardware and software systems.

### Kinematics and Dynamics Modeling

**Figure 2:**
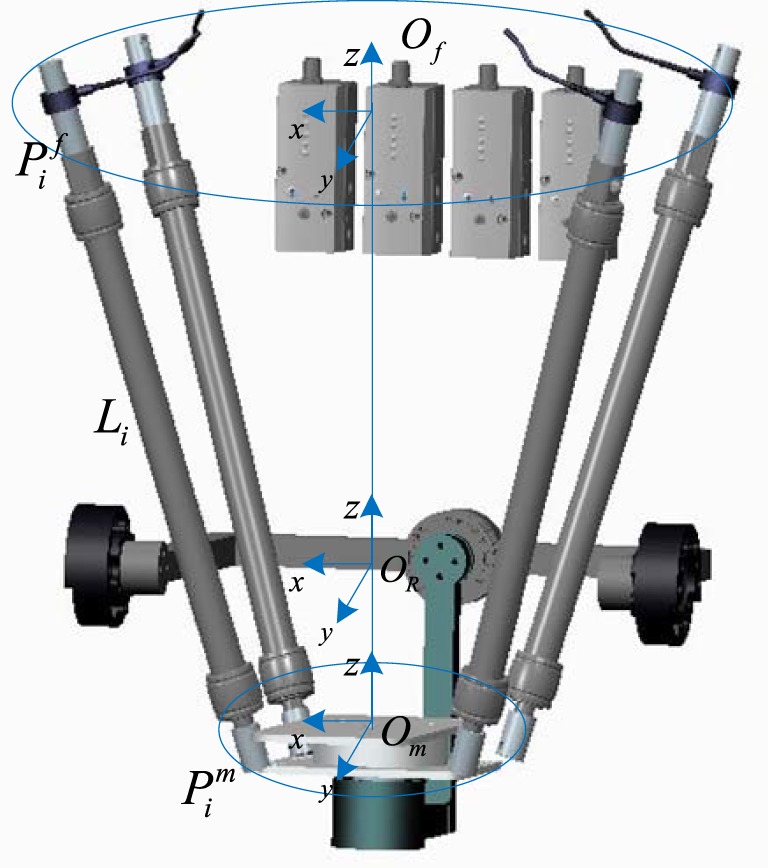
Geometric model the developed soft ankle rehabilitation robot (ARBOT). Define O_f_ is coordinate of fixed platform and the rotation center of end effector is denoted by O_m_, ${\text{P}}_{\text{i}}^{\;\text{f}}$ and ${\text{P}}_{\text{i}}^{\;\text{m}}$ are the connection point vectors of the upper fixed platform and the lower moving platform, respectively.

The Jacobian matrix relates the link velocities and the twist of the end effector, also the joint forces with the end effector torque, which is extensively used for kinematics and dynamic analysis. To get the Jacobian matrix, inverse kinematics of the robot must be modeled. Geometric model of the developed robot is illustrated in Figure 2 (Zhang et al., 2017a). The vector of each active link ***L****_i_* can be calculated given a desired end effector orientation θ = [θ*_x_*; θ*_y_*; θ*_z_*]. The inverse kinematics can be modeled by using following Eqs 1–3, where ${\text{R}}_{m}^{f}$ is the rotational transformation matrix, *H* is the distance between the center of upper fixed platform and that of the lower movable platform. *C* denotes the cosine function and *S* is the sine function, i.e., *C_y_* = cosθ*_y_*:

(1) $$\left\{\begin{array}{l} {\text{P}}_{i}^{f}={\left[\begin{array}{ccc} x_{i}^{f} & y_{i}^{f} & 0 \end{array}\right]}^{T}\\ {\text{P}}_{i}^{m}={\left[\begin{array}{ccc} x_{i}^{m} & y_{i}^{m} & 0 \end{array}\right]}^{T}\\ {\text{O}}_{\mathrm{fm}}={\left[\begin{array}{ccc} 0 & 0 & -H \end{array}\right]}^{T} \end{array}\right.$$

(2) $${\text{R}}_{m}^{f}=\left[\begin{array}{ccc} C_{z}C_{y} & -S_{z}C_{x}+C_{z}S_{y}S_{x} & S_{z}S_{x}+C_{z}S_{y}C_{x}\\ S_{z}C_{y} & C_{z}C_{x}+S_{z}S_{y}S_{x} & -C_{z}S_{x}+S_{z}S_{y}C_{x}\\ -S_{y} & C_{y}S_{x} & C_{y}C_{x} \end{array}\right]$$

(3) $${\text{L}}_{i}={\text{O}}_{\mathrm{fm}}+{\text{R}}_{m}^{f}{\text{P}}_{i}^{m}-{\text{P}}_{i}^{f}.$$

The length of each link ***l_i_*** can be calculated by ${\text{l}}_{i}=\sqrt{{{\text{L}}_{i}}^{T}{\text{L}}_{i}}$, so the element of Jacobian matrix ***J***_4×3_:

(4) $${\text{J}}_{i,j}=\frac{\partial l_{i}}{\partial \theta_{j}}=\frac{1}{l_{i}}{\left(\frac{\partial {\text{R}}_{m}^{f}}{\partial \theta_{j}}{\text{P}}_{i}^{m}\right)}^{T}\left({\text{O}}_{\mathrm{fm}}-{\text{P}}_{i}^{f}\right).$$

The relation between link velocities $\dot{\text{l}}$ and the end effector twist velocity **ω** can be expressed by Eq. 5, while the joint forces ***F*** can be mapped with the generated end effector torque **τ** by Eq. 6:

(5) $$\begin{array}{ll} {\dot{\text{l}}}_{4\times 1} & ={\text{J}}_{4\times 3}\omega_{3\times 1},\text{where}\dot{\text{l}}=\left[{\dot{l}}_{1};{\dot{l}}_{2};{\dot{l}}_{3};{\dot{l}}_{4}\right]\;\;;\\ \omega & =\left[\begin{array}{ccc} C_{y}C_{z} & -S_{z} & 0\\ C_{y}S_{z} & C_{z} & 0\\ -S_{y} & 0 & 1 \end{array}\right]{\left[\begin{array}{ccc} {\dot{\theta }}_{x} & {\dot{\theta }}_{y} & {\dot{\theta }}_{z} \end{array}\right]}^{T} \end{array}$$

(6) $$\tau_{3\times 1}={\text{J}}_{4\times 3}^{T}{\text{F}}_{4\times 1},\text{where}\;\tau =\left[\tau_{x};\tau_{y};\tau_{z}\right],\text{F}=\left[F_{1};F_{2};F_{3};F_{4}\right].$$

In this context, the end effector torque **τ** that the robot should overcome to reach a desired position **θ** can be calculated by using the dynamic model expressed by:

(7) $$\text{M}\left(\theta \right)\ddot{\theta }+\text{C}\left(\theta ,\dot{\theta }\right)\dot{\theta }+\text{G}\left(\theta \right)=\tau .$$

In which

(8) $$\text{M}\left(\theta \right)=\text{I}\left(\theta \right)\text{R}\left(\theta \right),\text{C}\left(\theta ,\dot{\theta }\right)=\text{I}\left(\theta \right)\dot{\text{R}}\left(\theta \right)+\tilde{\omega }\text{I}\left(\theta \right)\text{R}\left(\theta \right)$$

$\text{R}\left(\theta \right)=\left[\begin{array}{ccc} C_{y}C_{z} & -S_{z} & 0\\ C_{y}S_{z} & C_{z} & 0\\ -S_{y} & 0 & 1 \end{array}\right]$, I(θ) is moment of inertia matrix of the end effector and $\tilde{\omega }$ is a skew symmetric matrix of **ω**. In a scenario where the robot is applied to rehabilitation, the interaction torque between the robot and the human must be included in the dynamic model. The combined dynamic model of the patient–robot system is given by Eq. 9:

(9) $$\text{M}\left(\theta \right)\ddot{\theta }+\text{C}\left(\theta ,\dot{\theta }\right)\dot{\theta }+\text{G}\left(\theta \right)=\tau_{\mathrm{rob}}+\tau_{\mathrm{hum}},$$

where $\theta ,\dot{\theta },\ddot{\theta }$ are vectors of end effector angular position, velocity, and acceleration, respectively. ***M***(**θ**) is the mass matrix of robot end-effector. $\text{C}\left(\theta ,\dot{\theta }\right)$ is the calculated centrifugal and Coriolis torque, and ***G***(**θ**) is the gravity vector. **τ**_*rob*_ is the vector of torque generated by the robot and **τ**_*hum*_ represents the interaction torque applied by the human subject to the footplate.

## Hierarchical Compliance Controller

Different from rigid robots that can only provide “virtual” compliance when interacting with the environment, the PMAs-driven ones can provide variable inherent compliance during operations, which is able to make the robot being “real” soft. In this section, a hierarchical compliance control structure, including joint compliance adaptation and task admittance controller is developed.

### Robot Inherent Compliance Adaptation

Stiffness of a PMA can be regarded as the output force caused by unit changes of its length, which is adjustable (Meng et al., 2017b), so the robot inherent compliance can be adapted and this section will investigate how. Modeling of the robot with PMAs was a challenging task as the PMAs exhibit highly nonlinear and dynamic behaviors. In this study we considered Sarosi’s model developed in Sarosi et al. (2015). The function approximation method is appropriate for modeling robots actuated by PMAs and is adopted here to derive the PMA contraction force *F* based on its pressure inside *p* and contraction strain ε, as shown in Eq. 10, in which the strain is expressed as the ratio of the contraction length to the initial length. In this research, the parameters *a,b,c,d,e* were experimentally obtained by changing the muscle length while the contraction force and pressure were recorded (Zhang et al., 2015). Specifically, for inflation and deflation processes of the FESTO muscle, the model can be expressed by Eq. 11:

(10) $$F\left(p,\varepsilon \right)=\left(p+a\right)e^{b\cdot \varepsilon }+c\cdot p\cdot \varepsilon +d\cdot p+e$$

(11) $$\left\{\begin{array}{l} F_{\text{inf}}=\left(p+232.89\right)e^{-38.32\varepsilon }-904.01p\varepsilon +294.86p-289.06,\;\text{if}\;\text{inflation}\\ F_{\text{def}}=\left(p+272.70\right)e^{-32.58\varepsilon }-908.24p\varepsilon +298.83p-262.85,\;\text{if}\;\text{deflation.} \end{array}\right.$$

The vector of contraction forces generated by the four PMAs is: ***F***_4×1_ = [*F*_1_;*F*_2_;*F*_3_;*F*_4_]. The stiffness performance of the robot end effector ${\text{K}}_{\text{rob}}=\left[K_{\text{rob}}^{x};K_{\text{rob}}^{y};K_{\text{rob}}^{z}\right]$ can be expressed by Eq. 12, in which **τ**_rob_ is the function of ***J*** and ***F***. From Eq. 4, we know that ***J*** will be determined by the robot angle **θ**, and in Eq. 10 ***F*** is the function of muscle pressure *p* and the strain ε which is also dominated by the robot angle **θ**. Equations 10–12 indicate that the robot stiffness has a close relation with the pressure inside each muscle. The compliance property of the robot can be adapted by regulating the nominal pressure of the actuation muscles for their operating ranges. Unlike traditional 1-DOF joint actuated by antagonistic muscles, the developed robot is driven by four muscles in parallel, which means the torque-stiffness and pressure relations are not as simple as the previously presented equations such as those in Choi and Lee (2010) and Hussain et al. (2013c). In this research, we utilized experimental function approximation method to establish the relationship between robot end effector torque and angle and then derive the model for stiffness of the robot actuated by PMAs. In this way, the robot stiffness ***K***_rob_ can be obtained as a polynomial function of the nominal pressures *p*, as in Eq. 13. $k_{p}^{x}$, $k_{p}^{y}$ are the coefficients, superscript *x,y* represent the parameters are in ankle DP and IE, respectively. Then the robot compliance can be obtained from the estimated stiffness by ${\text{C}}_{\text{rob}}={\text{K}}_{\text{rob}}^{-1}$:

(12) $${\text{K}}_{\text{rob}}=\frac{\partial \tau_{\text{rob}}}{\partial \theta }=\frac{\partial {\text{J}}_{\left(\theta \right)}^{\text{T}}{\text{F}}_{\left(p,\theta \right)}}{\partial \theta }$$

(13) $$\left\{\begin{array}{l} K_{\text{rob}}^{x}\left(p\right)=k_{p1}^{x}p^{3}+k_{p2}^{x}p^{2}+k_{p3}^{x}p+k_{p4}^{x}\\ K_{\text{rob}}^{y}\left(p\right)=k_{p1}^{y}p^{3}+k_{p2}^{y}p^{2}+k_{p3}^{y}p+k_{p4}^{y}. \end{array}\right.$$

**Figure 3:**
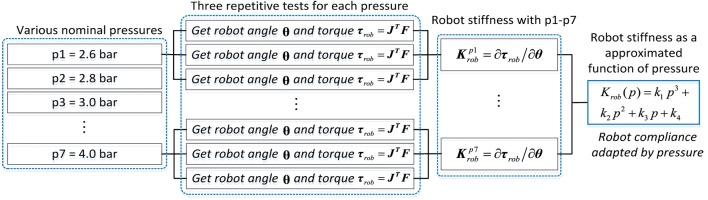
Diagram of the robot compliance modeling estimation and adaptation process.

**Figure 4:**
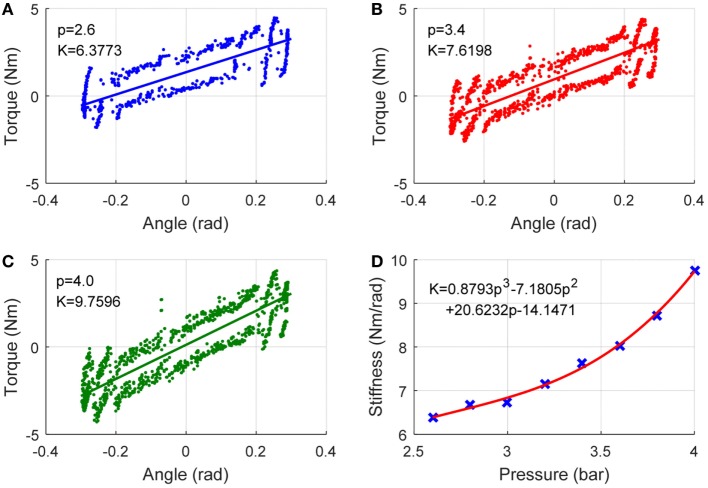
Robot inherent compliance adapted by nominal pressure about *X* (DP) axis. **(A–C)** the robot toque vs angle (to get stiffness ${\text{K}}_{\text{rob}}^{\text{x}}$) under three different pressures and **(D)** robot stiffness as a function of pressure, where the crosses denote seven pairs of robot stiffness vs pressure.

**Figure 5:**
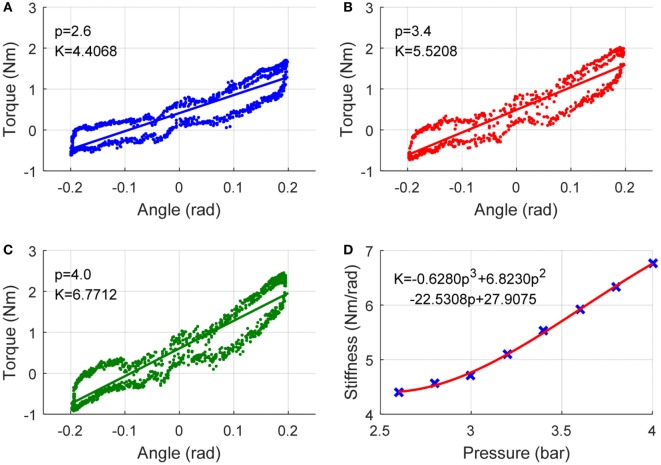
Robot inherent compliance adapted by nominal pressure about *Y* (IE) axis. **(A–C)** the robot toque vs angle (to get stiffness ${\text{K}}_{\text{rob}}^{\text{y}}$) under three different pressures and **(D)** robot stiffness as a function of pressure, where the crosses denote seven pairs of robot stiffness vs pressure.

The modeling and adaptation process of the robot inherent compliance can be demonstrated by the following diagram in Figure 3. Three repetitive tests will be conducted to gather the produced robot torque **τ**_rob_ and the robot movement trajectory **θ** data with different nominal pressures. The **τ**_rob_ is obtained from the real-time measured pulling forces ***F*** by using Eq. 6, and the stiffness ***K***_rob_ is then calculated using Eq. 12. To reach an appropriate three-order function approximation to fit the curve of stiffness and pressure, seven trials were performed and seven pairs of pressure *p* and stiffness ***K***_rob_ were collected. The robot was controlled at low speeds during the experiment to avoid the effect of inertial torques and forces so as to produce pure stiffness. The adopted PMAs can work normally with a maximum pressure of 6.0 bar. Here, a sequence of experiments with pressures range from 2.6 to 4.0 bar (i.e., 2.6, 2.8, 3.0, 3.2, 3.4, 3.6, and 4.0 bar) were performed. Figures 4 and 5 demonstrate the obtained experimental results about Euler *X-* and *Y* -axis, respectively. Here, three examples with pressure 2.6, 3.4, and 4.0 bar are given, and the corresponding generated end-effector torques and estimated stiffness parameters are shown in (a)–(c) of Figures 4 and 5. After seven trials, the robot stiffness ***K***_rob_ is fitted as a function of nominal pressure *p*, as shown in (d) of Figures 4 and 5. Consequently, the robot stiffness in Euler *X*-axis can be expressed by $K_{\text{rob}}^{x}\left(p\right)=0.8793p^{3}-7.1805p^{2}+20.6232p-14.147$ and the same procedure is conducted on Euler *Y* -axis, then $K_{\text{rob}}^{y}\left(p\right)=-0.628p^{3}+6.823p^{2}-22.5308p+27.9075$.

### End-Effector Admittance Control

Admittance control of the robot is a well-established method to modify the robot compliance behavior and assistance according to the active torque applied by the subject. The admittance controller of the ARBOT is implemented in task space, as in Eq. 14. Define **θ***_d_* the initial desired trajectory of the robot, and it will be changed to the new reference trajectory **θ***_r_* after an active ankle torque applied by the subject. Three rotary joint encoders have been used to measure the actual ankle-robot angular trajectories **θ***_a_* in DP, IE and AA directions:

(14) $${\text{M}}_{d}\left({\ddot{\theta }}_{r}-{\ddot{\theta }}_{d}\right)+{\text{B}}_{d}\left({\dot{\theta }}_{r}-{\dot{\theta }}_{d}\right)+{\text{K}}_{d}\left(\theta_{r}-\theta_{d}\right)=\tau_{\text{act}}.$$

The six-axis load cell is used to measure the interaction torque **τ**_mea_ between the robot and the human subject. Note that the human–robot interaction torque provided by the load cell includes both active ankle torque and passive torque that contains gravitational and inertial components (Hussain et al., 2013c). The active torque component **τ**_act_ is generated by the human voluntary interaction, while the passive torque comes from other components generated by skeletal muscles and viscoelastic components such as ligaments and other tissues surrounding joints, which can be written as **τ**_pas_:

(15) $$\tau_{\text{mea}}=\tau_{\text{act}}+\tau_{\text{pas}}.$$

**Figure 6:**
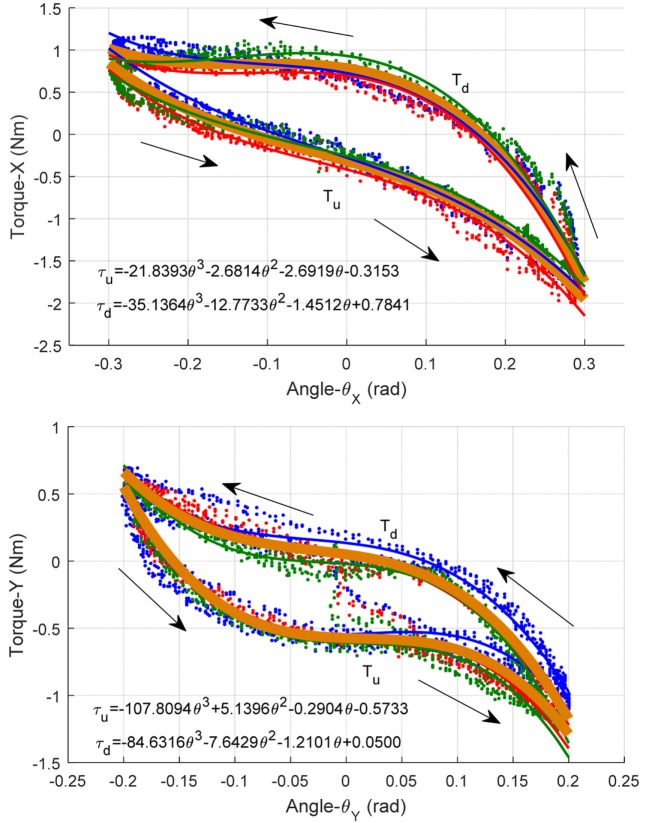
Human ankle passive torque estimation: modeling the torque as a function of ankle joint rotation angle. (Top) Passive ankle toque vs angle about DP and (bottom) about IE.

To obtain the human active ankle torque, the estimation of subject’s passive ankle torque on the robot should be modeled. In order to estimate the ankle torque, a dynamic experimental modeling method is used. Considering DP and IE degrees of freedom and the dynamic relation between the measured torque and the rotatory displacement, the model can be expressed by

(16) $$\left\{\begin{array}{l} \tau_{\text{pas}}^{x}\left(\theta \right)=k_{1}^{x}\theta_{x}^{3}+k_{2}^{x}\theta_{x}^{2}+k_{3}^{x}\theta_{x}+k_{4}^{x}\\ \tau_{\text{pas}}^{y}\left(\theta \right)=k_{1}^{y}\theta_{y}^{3}+k_{2}^{y}\theta_{y}^{2}+k_{3}^{y}\theta_{y}+k_{4}^{y}, \end{array}\right.$$

where θ*_x_* and θ*_y_* are the DP and IE angular position, respectively, and $k_{i}^{x}$ and $k_{i}^{y}$ are corresponding coefficients. $\tau_{\text{pas}}^{x}$ and $\tau_{\text{pas}}^{y}$ are the torques generated by the ankle passive movement. For the purpose of estimating the ankle-robot passive torque for a specific subject, the participant will be asked to keep passive during the robot operation, a slow sine wave is adopted as the reference trajectory and the measured torque and angular displacement are recorded, respectively. To remove the differences of nominal pressures on the passive torque, three levels of pressures are adopted and the data from each test are plotted. The data including ankle joint angle θ and the measured passive torque τ_pas_ will be collected from three repetitive tests to estimate the relationship between them. By averaging the interaction torques-angles of all tests, a reference function of torque-angle profile representing the estimated passive torque was calculated, as shown in Figure 6.

In this figure, the blue, red, and green spots are the collected ankle passive torque-angle data pairs measured under three different PMA pressures of 2.6, 3.4, and 4.0 bar, respectively. The three thin lines are their fitted toque-angle functions, which have no significant difference actually. This indicates that the human ankle passive torque measured by the robot is independent with the PMA pressure, and it is in consistent with our assumption that the passive torque is largely determined by the human muscle and ligament conditions. Then the overall relationship between the estimated ankle passive torque and the rotation angle is approximated using all collected experimental data by a three-order polynomial function, which is illustrated by a thick orange line in Figure 6. Note that the passive torque will be different when the ankle is moving toward different directions. When the robot is driving the ankle move toward positive *X* and *Y* directions, we note the passive torque as τ_u_, in contrast, the torque toward negative directions is noted as τ_d_.

To understand participant’s voluntary efforts, the passive torque model needs to be separated from the active torque as well as the robot gravitation and inertia during operation. As the aim of robot-assisted active training is to allow robot to respond to active contributions of the human subject, by isolating the passive components the controller can avoid robot responding to passive effects.

Then, the active ankle torque component $\tau_{\text{act}}=\left[\tau_{\text{act}}^{x};\tau_{\text{act}}^{y};\tau_{\text{act}}^{z}\right]$ can be extracted from the measured human interaction torque **τ**_mea_ and the passive effects **τ**_pas_. In *X* and *Y* axes:

(17) $$\left[\begin{array}{c} \tau_{\text{act}}^{x}\\ \tau_{\text{act}}^{y} \end{array}\right]=\left[\begin{array}{c} \tau_{\text{mea}}^{x}\\ \tau_{\text{mea}}^{y} \end{array}\right]-\left[\begin{array}{c} \tau_{\text{pas}}^{x}\\ \tau_{\text{pas}}^{y} \end{array}\right]$$

By measuring the active torque estimate applied by the user, it is possible to compute the new reference trajectory required to make the robot operate in certain mass, stiffness, and damping parameters. As a result, the adapted angle **θ***_r_* can be obtained with an admittance filter as

(18) $$\theta_{r}=\frac{\tau_{\mathrm{act}}}{{\text{M}}_{d}s^{2}+{\text{B}}_{d}s+{\text{K}}_{d}}+\theta_{d}.$$

### Adaptive Control of Hierarchical Compliance

The robot end effector and actuator stiffness is the key factor to influence its compliance. As the dynamics of the human ankle are not precisely known, the stiffness value of the admittance controller should be selected with certain assumptions to obtain a desired system response. The concept behind adaptive compliance control is to set the robotic compliance low (impedance high) if the human subjects present little effort or participation. Such a low compliance will increase the robotic assistance. Similarly, the compliance of the robot will be adapted to a high level (impedance low) if a greater effort is detected from the subjects. Such a high compliance will allow the subject to have more active freedom during movement training. In practice, the measurement of interaction force and movement can be used to assess the quantitative level of motor function recovery (Hussain et al., 2013c).

**Figure 7:**
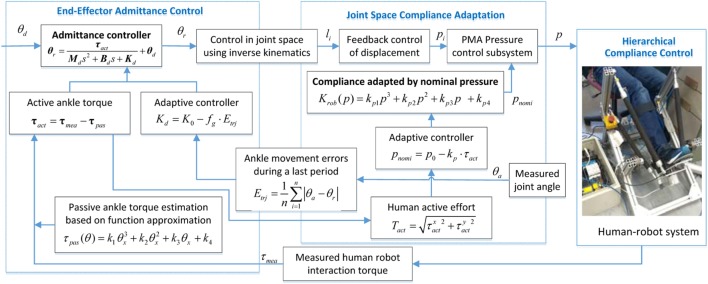
Adaptive hierarchical compliance controller for the ankle rehabilitation robot (ARBOT).

In this paper, we propose a two-level strategy to adapt the robot compliance according to the human behavior, assisting the subject to perform the best effort to successfully complete the task imposed by the game. The robot compliance in joint and task space are both adapted online based on the subject’s active torque estimate and the movement ability assessment. The general structure of the adaptive compliance controller is shown in Figure 7. By taking into account both joint space stiffness control based on nominal pressure adjustment and the task space admittance control, the vector of compliance control parameters for the robot is defined as ***v*** = [*v*_1_; *v*_2_], in which *v*_1_ is the mean nominal pressure of the four pneumatic muscles, while *v*_2_ is the admittance parameters (stiffness here) of the robot end effector when it interacts with the human ankle joint.

The controller is providing adapted admittance in task space by taking into account the subject’s movement ability and adjusting the compliance level of the robot itself in joint space by considering the human active ankle torque. This is achieved by directly influencing the nominal pressure of each actuator which determines the compliance property of the robot. The objective of joint space compliance control law is to adjust the pneumatic muscle’s pressure according to the human subjects’ active contribution so that the joint stiffness can be adjusted dynamically. And, the robot stiffness will, in turn, increase or decrease the amount of joint force and also the robot output (**τ**_rob_).

The first compliance control strategy is based on the necessary actuator stiffness that the robot system should achieve to complement the subject’s own active efforts. To this end, we reduce the robot assistance output when the subject contributes more efforts. It is known from Eq. 13 that the robot stiffness ***K***_rob_ is determined by the nominal pressure inside pneumatic actuators, and since the stiffness can be expressed as the assistance torque per move unit as in Eq. 12, so a higher nominal pressure produces a larger stiffness and also implies a higher assistance level. In order to adjust the robot assistance output, the nominal pressure inside should be adapted online by monitoring the subject’s active participation and this process can be expressed by the equation below:

(19) $$p_{\text{nomi}}=\left\{\begin{array}{ll} p_{\text{min}}\; & \text{if}p_{\text{nomi}}<p_{\text{min}}\\ p_{0}-k_{p}\cdot T_{\text{act}}\; & \text{if}p_{\text{min}}\leq p_{\text{nomi}}\leq p_{\text{max}}\\ p_{\text{max}}\; & \text{if}p_{\text{nomi}}>p_{\text{max}}, \end{array}\right.$$

where *p*_0_ is the initial nominal pressure, *k*_p_ is a positive factor to reduce the robot assistance when encountering high active effort. *p*_nomi_ is the introduced nominal pressure that can be set to *p*_min_ to achieve the maximum actuator compliance and increased to *p*_max_ to increase the stiffness and provide more robot assistance, working at a saturation function to guarantee the applied pressure is in a reasonable range from *p*_min_ to *p*_max_. All the parameters are determined by experimental trials and can be tuned to suit specific subjects to satisfy their training needs. For example, the initial pressure *p*_0_ for a healthy subject is set low to ensure compliance while can be higher for a patient, and the coefficient *k_p_* can be set large to achieve a quick response while a smaller *k_p_* can provide more stable assistance. To realize unlimited assistive behavior of the soft ankle robot, high actuator compliance is beneficial, but in case of too high or too low stiffness, system stability must be provided by the saturation function. It adjusts compliance behavior of the robot actuator and will reduce the supportive nominal pressure when active torque is large to motivate the subjects to contribute more their own efforts during the robot-assisted training. The compliance adaptation law given above can be expressed as a combination of human active effort (*T*_act_) to a saturation function sat, and the *T*_act_ should reflect the human activities in both DP and IE directions:

(20) $$T_{\text{act}}=\sqrt{{\tau_{\text{act}}^{x}}^{2}+{\tau_{\text{act}}^{y}}^{2}}.$$

The joint space compliance controller directly influence the robot inherent compliance based on the human active ankle torque, while the second compliance control strategy aims to minimize the task reaching errors while minimizing the robot efforts to characterize the assist-as-needed paradigm. The admittance of the robot in task space is also online adapted to the individual’s behavior and ability, providing adaptive compliance level and support assistance. This scheme attempts to increase the robot compliance and allow for a higher freedom while achieving an admissible target error. When the subject shows better movement ability, the robot system should be more compliant to allow a higher free deviation. We present an error-based estimation for task complete level to assess the subject’s movement ability, which is a performance-based adaptation strategy

(21) $$K_{\text{adm}}=\left\{\begin{array}{ll} K_{\text{min}}\; & \text{if}K_{\text{adm}}<P_{\text{min}}\\ K_{0}-f_{g}\cdot E_{\mathrm{trj}}\; & \text{if}K_{\text{min}}\leq K_{\text{adm}}\leq K_{\text{max}}\\ K_{\text{max}}\; & \text{if}K_{\text{adm}}>K_{\text{max}}, \end{array}\right.$$

where *E*_tri_ is the ankle movement errors that can reflect the subject’s movement ability, which is online learning obtained during a previous certain period of time, as in Eq. 22. *K*_0_ is the initial base admittance parameter, *f_g_* is a positive factor to ensure that the deviation freedom be reduced when encountering high errors. Similarly, these parameters will be decided by trials to guarantee that the robot behaves safely and responds its admittance to the movement errors in a certain speed. More details on the parameters selection and determination will be discussed in the experiment part:

(22) $$E_{\text{trj}}=\frac{1}{n}\sum_{i=1}^{n}\;\left|\theta_{a}-\theta_{r}\right|,$$

*n* is the sample number. This equation is used in the compliance control law, in order to raise the robot admittance level, i.e., a higher free deviation, when errors are small, to prevent patients from relying on the robot. By contrast, when errors are large, the robot admittance will decrease, resulting in a higher impedance level that allows the robot to provide more assistance to guide the patient’s movement. The reason behind this adaptation law is to motivate the patients to maximum their voluntary physical efforts. The similar saturation and linear adaptation rules are adopted for the end effector compliance control. If the subject has a good task result during the last n/20 s (*E*_trj_ is small), the robot admittance will increase due to the factor, resulting in the reduced robot assistance. On the other hand, if the subject has a bad performance, the robot assistance will increase. Overall, this hierarchical compliance adaptive control scheme will make the robot provide adaptive assistance for the individual participant’s ankle and will be online learning the participant’s behavior, performance and abilities so as to enhance the participant’s voluntary effort.

## Experiments and Results Discussion

**Figure 8:**
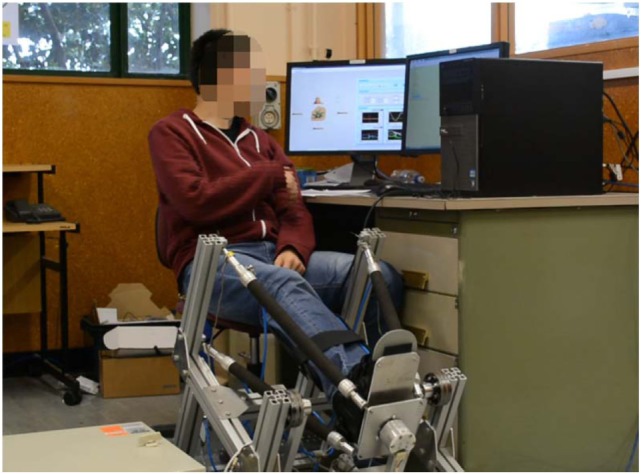
Experimental setup of the human-ankle robot system for compliance control tests. The participant had agreed by written informed consent for using his picture.

The proposed control schemes for robot hierarchical compliance adaptation were evaluated on a human-ankle robot system, as shown in Figure 8. Three healthy subjects noted as S1 (male, age 27, height 170 cm, weight 61 kg), S2 (male, 31, 178 cm, 75 kg), and S3 (female, 29, 165 cm, 52 kg), and one cerebral palsy (CP) patient with limited ankle movement ability (S4: male, 42, 180 cm, 87 kg) were involved in the experiment. Each participant was asked to sit on a height adjustable chair with the foot strapped to the end-effector. The goal of the experiments is to verify the general reaction of the compliance controller using the soft ARBOT to different subject’s behavior. The experiment protocol consists of the evaluation of two adaptive solutions, joint space pressure adjustment and task space admittance adaptation, which will be evaluated individually.

To applying on human subjects, various measures had been taken to guarantee the safety during operation. The PMAs are intrinsically compliant and the nominal pressure was controlled within a moderate range to make the robot soft and inherently safe for any interactions. The amplitude and speed of robot movement were controlled under a saturation function restricted to the movement ability of each recruited participant. Emergency stops had also been designed from both hardware and software aspects, which mean the robot can be stopped immediately at any time when the participants feel uncomfortable or it will stop automatically once the interaction torque reaches a threshold that exceeds the participant’s comfortable level. This trial has been approved by the Human Participants Ethics Committees from Wuhan University of Technology, China and University of Auckland, New Zealand, and written informed consent was obtained from each participant.

### Passive Control with Varying Robotic Compliance

A passive training control with circle movement along DP and IE axis was performed to show the influence of nominal pressure on the human–robot system’s inherent compliance. The reference trajectory was an ellipse with X amplitude of 0.22 rad and Y amplitude of 0.12 rad, and the robot was controlled for 80 s with a 0.05 Hz frequency. Four different levels of nominal pressure were used to vary the robotic inherent compliance: 0, 2.6, 3.4, and 4.0 bar. The subjects were asked to move within passive robot for 80 s in each reference trajectory tracking control. This experiment aims to show the relationship between robot nominal pressure and its compliance performance, results of participant S1 are presented and discussed in this section.

**Figure 9:**
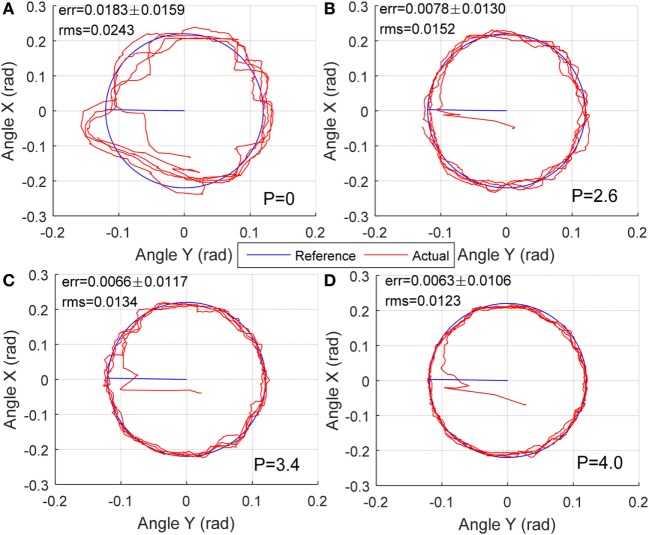
Human–robot passive movement training with varying compliance dominated by nominal different pressures: **(A)** *P* = 0, **(B)** *P* = 2.6 bar, **(C)** *P* = 3.4 bar, and **(D)** *P* = 4.0 bar.

The robot reaction to different nominal pressure is presented in Figure 9, shown are average errors and SDs of four experiments with same external conditions. The deviation levels were determined by calculating the Euclidian distance (error) between the measured position and the reference profile. The mean error and the root mean square (RMS) represent a quantitative evaluation whether the robot is more likely to be in a compliant state, with a large deviation or in a stiff state, with a small deviation. From Section “Robot Inherent Compliance Adaptation” we found that the robot stiffness became higher when the nominal pressure increased and, thus, the compliance was lower. So when the robot was fully compliant with 0 nominal pressure, the subject was completely free to move the end-effector along the reference trajectory; however, the average tracking error and RMS of error was the highest, i.e., 0.0183 and 0.0243 rad, respectively. As the increase of robot stiffness as well as the reduction of robot compliance, the subject ankle’s deviation freedom from the reference path became smaller, which can also be reflected by the better trajectory tracking results with errors of 0.0078, 0.0066, and 0.0063 rad for the three different levels of softness, respectively. At the same time, the reduced RMS of errors help conclude that the robot compliance is becoming lower and the robot assistance contribution is high. The subject in high compliance tests (low pressure) showed greater deviations from the reference target tracking trajectories as compared to the stiffness mode (high pressure) because the subjects were provided more freedom by the robot during the low pressure mode, i.e., the fully compliant mode. Here, we regulated the robot compliance only in joint space, without task space adaptation or motion change. From the results, we found that when a patient recovered from highly impaired to healthy, such a method is not able to provide a changeable training with adjustable robot interaction and variable challenging tasks that allow the patient to be more motivated during robot-assisted rehabilitation.

### Game-Guided Adjustable Admittance Control

**Figure 10:**
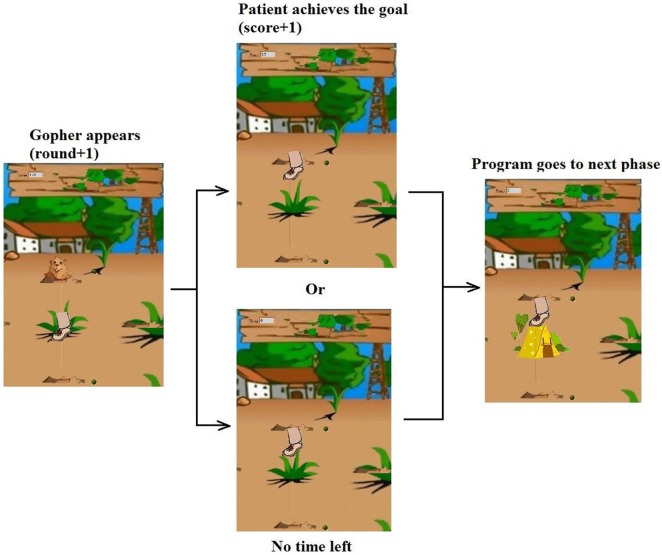
The virtual reality game designed to guide the patient perform target reach training.

Then, the game-based task space control was performed to show the influence of admittance levels on the human–robot system. In this experiment, we developed a set of virtual reality game for subjects in seated position (Figure 8) to guide the subject to perform ankle movements, mainly the DP and IE here. The developed games target to achieve a sequence of gophers that appear randomly on two sides of the screen (vertical to DP and horizontal to IE), see Figure 10. The subject must hold the foot (represented the robot orientation) in the interactive environment, and then drive the ankle to hit the desired target, e.g., the gopher in Figure 10. Once the target is reached, it is given time for movement back to the center and then appears a new target. The DP targets are set 0.25 rad to the start position and the IE targets 0.15 rad. The goal is to encourage patients to make the maximum effort at each movement and reach the largest number of targets within a certain period of time. We reduced the robot assistance while playing visual reality game to motivate the patients contribute more during the ankle therapy. The subject was instructed to perform the target reaching game within 200 s for each session, and a 5-min rest time is given to the subject between sessions. Four different levels of admittance were used: 0, 0.015, 0.03, and infinite.

**Figure 11:**
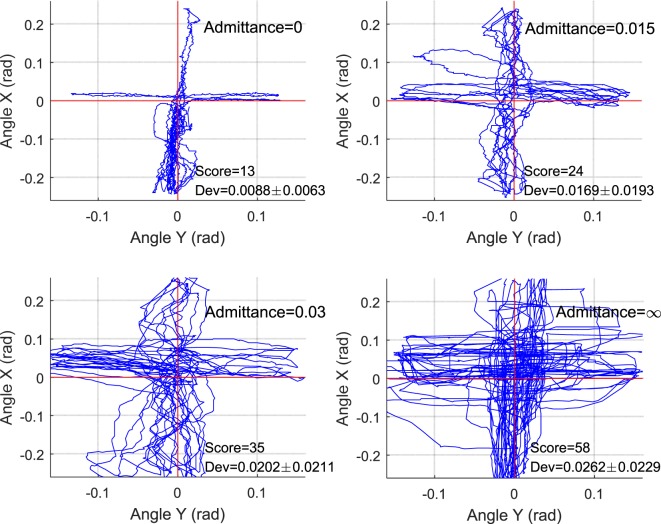
Target reach game-guided movement training results with adjustable end-effector admittance, in which red line the reference and blue is the actual trajectories.

In order to analyze the proposed strategies and the patient’s performance based on kinematic and kinetic data recorded during gaming, a set of parameters were defined for each successful move, i.e., how many targets are successfully reached and how the actual trajectory deviated from the reference one. Experiment results of S1 are also presented here to show the relationship between robot admittance level and the participant’s movement freedom performance. The experimental results of the subject with four different admittance parameters are shown in Figure 11, in which the achieved scores and the actual ankle movement trajectories as well as their deviations during the training are plotted. The results showed that when the robotic end-effector admittance was set 0, the ankle movement was strictly restrict to the reference path, so the deviation was the smallest but the score was also low due to the robot stiffness where participant cannot speed up the robot movement. With the increase of admittance from 0.015 to 0.03, the participant received more freedom and contributed more efforts to the training game tasks, so the deviations grew and the score became higher. Finally, the participants were set fully free with infinite admittance to stimulate the completely recovered patient and in this case the score was the highest but the deviation was also large. This game-guided control with relatively high admittance can further motivate the patient’s intention to contribute by reaching more game targets. Notice that the IE movements were more difficult to perform accurately than the DP, as greater deviations were found in this axis due to the gravitational effects. In this experiment, we tested the task space compliance controller, which only adjusted the end-effector admittance. From results in Figure 11, we can see that the task complete performance, i.e., the movement accuracy, cannot be guaranteed without proper assistance force, which should be regulated by taking into account the joint compliance property.

### Adaptive Hierarchical Compliance Control

Finally, the above two compliance solutions were integrated and tested as a whole game-guided training system. Experiments with three healthy subjects (S1, S2, and S3) and one CP patient (S4) were conducted to evaluate if the proposed compliance control scheme could adjust the robotic stiffness and assistance according to the active participation and movement ability of the subjects. The participant’s active participation can be indicated by the calculated active ankle torque and the movement ability represented by the trajectory deviation errors. The experiment was performed to test the hierarchical compliance control scheme from patient highly inactive to highly active modes. Each participant was trained several times for familiarization before the final recorded experiment. During the tests, each subject’s active torque and movement ability will be assessed (Zhang et al., 2016) and based on trial and error the control parameters will be determined in experimental manner to ensure that robot can provide specific training needs and compliance for an individual. *p*_0_ and *K*_0_ are base values, *k_p_* and *f_g_* are weighting coefficients that can be used to adjust the influence of human active effort and movement performance on the robot compliance. For example, *p*_0_ can be set higher for a subject who needs more assistance from the robot, while a smaller *f*_g_ is suitable for an highly active subject as it can make the task more challenging and keep the interaction stable. In this experiment, the parameters of each subject were set as follows: (S1) *p*_0_ = 4, *k_p_* = 0.4, *K*_0_ = 0.025, *f*_g_ = 0.3; (S2) *p*_0_ = 4, *k_p_* = 0.35, *K*_0_ = 0.025, *f_g_* = 0.25; (S3) *p*_0_ = 3.8, *k_p_* = 0.4, *K*_0_ = 0.03, *f_g_* = 0.3; and (S4) *p*_0_ = 4.3, *k_p_* = 0.45, *K*_0_ = 0.025, *f_g_* = 0.35. All parameters were adjusted within a constrained space to guarantee the system stability. During the trials, subjects were instructed to increase their active contribution every 100 s at each time, totally 400 s, noted as phase A (0–100 s), phase B (100–200 s), phase C (200–300 s), and phase D (300–400 s), respectively. The nominal pressure was online adjusted by the immediate active ankle torque and the admittance parameter was tuned by the online learning of the subject’s movement errors within the last 5 s (sample number for evaluation *n* = 100). The objective of change from inactive to active is to determine if the controller can reduce the robot output and increase its compliance so that more voluntary freedom is allowed for the subjects.

**Figure 12:**
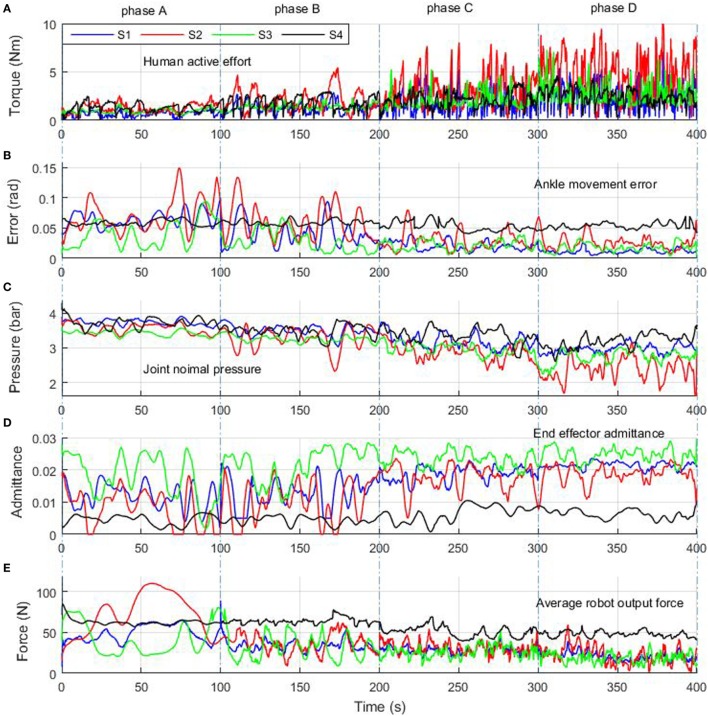
Experiment results of adaptive hierarchical compliance control with four human subjects who were asked to increase active contribution each time from phase A to phase D. Human active effort **(A)** was calculated using Eq. 20, ankle movement error **(B)** was obtained from using Eq. 22, joint nominal pressure **(C)** was regulated based on human participation by Eq. 19, end-effector admittance **(D)** was adjusted to human movement error (Eq. 21), and average robot force **(E)** was the mean value of four muscles’ pulling forces. Blue, red, green, and dark lines represent the results of S1, S2, S3, and S4, respectively.

Preliminary results of the adaptive compliance control scheme developed for the robotic ankle training are presented in Figure 12, which shows typical responses of each subject’s active torque estimate, calculated movement error, nominal pressure, and robot admittance level, as well as the robot link force. The first plot (Figure 12A) shows the changes of human active torque and the second plot (Figure 12B) shows the movement errors calculated based on the absolute value of trajectory difference. Both used as input for the adaptive compliance controller. The third plot (Figure 12C) shows the controlled actuator nominal pressure and the fourth (Figure 12D) shows the adjustment of robot admittance in task space. During the first 100 s with highly inactive mode (phase A), all subjects exerted small active efforts and the large movement errors were caused by different passive effects, so the control scheme adjusted the robot compliance to a lower level, which ensured that the robot is capable of guiding the subjects’ limbs on reference trajectories and providing enough assistance. From phase B to C, the three healthy subjects showed dramatic changes in the active efforts and movement errors. The patient also had a slightly increased active torque when he was asked to take more efforts. During the last 100 s when the subjects contributed more actively (phase D), they were able to perform the task with a satisfactory performance, and the control scheme set the robot compliance to a high level, indicated by the lower pressure and higher admittance. This variation in robotic compliance demonstrates the adaptive controller’s ability in regulating the robotic compliance according to the subject’s active participation and movement ability. The average pneumatic muscle contraction force applied by the robot during the adaptive compliance control experiment is shown in Figure 12E to demonstrate the regulations of robot assistance outputs.

In a rehabilitation process, the most suitable human cooperative results are achieved when the robot aims to reach the best movement performance while making the subject’s voluntary effort maximum and the robot output minimum (Ibarra et al., 2014). It can be seen from Figure 12 that once the subjects started behaving actively during the movement, the robot-generated joint pulling forces decreased, which indicates that the proposed control scheme is capable of providing adjustable assistance according to the participant’s contributed active effort. The robot assistance decreases by applying a lower pressure and a higher admittance according to the increase of subject’s participation and decrease of movement error, following the Eqs 20–22. Statistical analysis of the experimental results is summarized in Table 1. Active effort dada of the three healthy subjects show that they increased their participation gradually from phases A to D, and during this period the movement errors were all reduced steadily. This information show that when the subjects were instructed to perform from highly impaired to healthy, the robot system can monitor such recovery changes and the robot was controlled to respond accordingly by reducing the robot assistance and raising the compliance with a decreased nominal pressure and higher admittance level. While for the patient, results also showed a slight improvement in the active efforts but very limited changes in the movement error (from 0.58 to 0.54 rad), so the robot kept a high stiffness level and stable impedance (admittance around 0.05) to provide more assistance to the patient during the training task. Even so we can see the control system has tried to adjust its inherent compliance (pressure changed from 3.73 to 3.19 bar) to the patient’s active interaction (1.29 to 2.52 Nm) to present the patient’s positive influence on the robot behavior and maximum his participation.

**Table 1:** Statistical results of the involved four subjects in different phases A–D.

Human ability robot output		Active effort (Nm)	Movement error (rad)	Game score	Deviation RMS	Nominal pressure	Admittance level	Robot force (*N*)
S1	Phase A	0.65 ± 0.29	0.064 ± 0.013	4	0.0399	3.74 ± 0.08	0.012 ± 0.004	48.96 ± 10.06
	Phase B	1.27 ± 0.56	0.041 ± 0.020	20	0.0374	3.50 ± 0.15	0.013 ± 0.005	34.30 ± 6.76
	Phase C	2.01 ± 1.14	0.021 ± 0.007	31	0.0273	3.20 ± 0.22	0.019 ± 0.002	28.93 ± 4.92
	Phase D	2.83 ± 1.28	0.012 ± 0.004	31	0.0161	2.97 ± 0.13	0.021 ± 0.001	20.73 ± 5.09

S2	Phase A	1.26 ± 0.50	0.071 ± 0.027	5	0.0453	3.55 ± 0.15	0.008 ± 0.005	72.63 ± 25.45
	Phase B	2.04 ± 1.12	0.059 ± 0.027	14	0.0429	3.28 ± 0.31	0.011 ± 0.006	37.01 ± 11.44
	Phase C	3.17 ± 1.56	0.030 ± 0.016	26	0.0309	2.88 ± 0.24	0.017 ± 0.004	31.56 ± 7.22
	Phase D	4.62 ± 2.13	0.026 ± 0.011	25	0.0228	2.38 ± 0.30	0.019 ± 0.003	22.43 ± 10.71

S3	Phase A	0.94 ± 0.24	0.040 ± 0.023	5	0.0400	3.42 ± 0.08	0.018 ± 0.007	41.86 ± 19.91
	Phase B	1.36 ± 0.32	0.028 ± 0.017	12	0.0321	3.25 ± 0.09	0.022 ± 0.005	32.54 ± 15.49
	Phase C	2.02 ± 0.82	0.022 ± 0.008	24	0.0283	2.98 ± 0.17	0.024 ± 0.002	26.30 ± 7.10
	Phase D	2.66 ± 1.06	0.018 ± 0.007	25	0.0211	2.74 ± 0.19	0.025 ± 0.002	19.03 ± 5.91

S4	Phase A	1.29 ± 0.44	0.058 ± 0.005	6	0.0334	3.73 ± 0.14	0.004 ± 0.002	62.82 ± 3.91
	Phase B	1.83 ± 0.67	0.059 ± 0.004	10	0.0290	3.49 ± 0.18	0.004 ± 0.001	64.08 ± 3.76
	Phase C	2.07 ± 0.76	0.053 ± 0.008	11	0.0276	3.37 ± 0.21	0.006 ± 0.002	50.97 ± 6.70
	Phase D	2.52 ± 0.80	0.054 ± 0.005	13	0.0289	3.19 ± 0.20	0.006 ± 0.001	48.10 ± 3.62

*Active effort and movement error (mean value and SD), game score and movement deviation indicate partial human ability during each phase, while the nominal pressure, admittance level, and averaged muscle pulling force (mean value and SD) show the robot output in each phase*.

**Figure 13:**
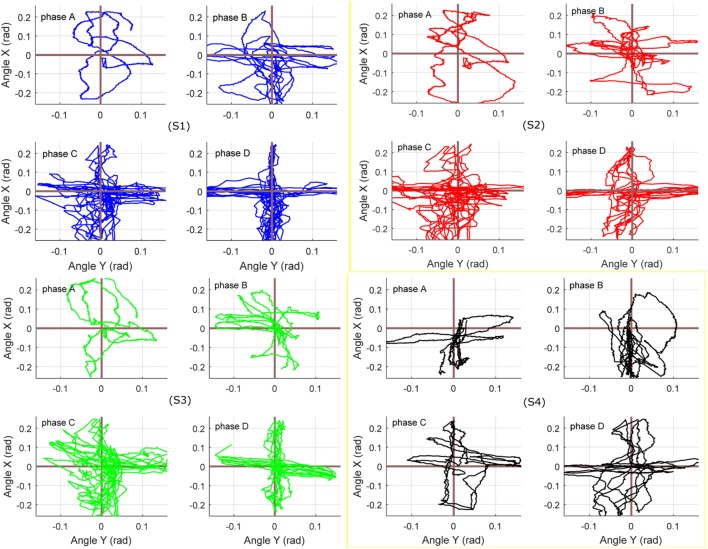
Target reaching game results with hierarchical compliance control: the figures of four subjects from phases A to D indicate that the robot can cooperate to achieve better and better performance. Blue, red, green, and dark lines represent the results of S1, S2, S3, and S4, respectively.

To study the differences for each separate control mode from highly inactive (phase A) to highly active (phase D), Figure 13 shows the movement performance of each subject during the game training. The robot-ankle angular trajectory during each 100 s from phases A to D was recorded and the game score and deviation extent (RMS) was also assessed in Table 1. All subjects sustained a low performance during phase A, e.g., averaging over 0.06 rad error and score 4 measured from S1, thus the robot assistance level was high. In comparison, for the last 100 s in phase D, the good movement performance (S1’ error just over 0.01 rad and score 31) allowed the level of robot assistance, represented by the muscle actuator forces, to decease significantly. Though the patient only had limited active efforts, the robot could adjust its assistance and compliance to achieve a better and better result (a bit higher score and smaller deviation) from phases A to D. Overall, the statistical results of the hierarchical compliance control are encouraging with the robot assistance being adjusted to suit each subject’s needs the human–robot together completing the task. By regulating the pressure inside each PMA, the adaptive compliance controller is providing sufficient pressure for subjects who have no sufficient efforts and will reduce the pressure, i.e., increase the robot compliance, in case of higher active participation and the robot task space compliance also increases to consider the subject’s behavior and performance to maximize the human voluntary effort.

### Discussion

The paradigm of compliance control is important in rehabilitation field and there have been some extensive work such as the patient-cooperative or assist-as-needed strategy (Hussain et al., 2017; Zhang et al., 2017b), especially on Lokomat and LOPES (Fleerkotte et al., 2014; Meuleman et al., 2016). However, most of existing works are applied to traditional rigid robots. Adaptive interaction control strategies on compliant robots have just been investigated in recent years. A common method is based on the task space impedance control (Lerner et al., 2017). Hussain et al. (2013c) proposed an adaptive controller for a soft robotic gait orthosis to adjust the end-effector impedance according to the subject’s voluntary participation. Jamwal et al. (2016) also applied a similar task space compliance control strategy on an ARBOT driven by pneumatic muscles. Most of current studies only concentrated on the task space and did not take into account the pneumatic muscle’s compliance property. Limited work has been reported on the joint space control. Wilkening et al. (2015) proposed an adaptive assistive controller for a soft elbow trainer, in which the supportive pressure inside pneumatic bending joint was directly controlled. It indicated that the actuator stiffness would be influenced by the initial pressure but the relationship between them was not investigated or modeled. Choi and Lee (2010) presented a method to independently control the robot compliance and position, for a pneumatic muscles actuated joint. However, the relationship between pressure and robot stiffness was not explored, and the pressure-based compliance control have not been reported in the literature. In this paper, we have developed a compliance control scheme for the intrinsically CARR. This control scheme, including two control strategies, provides seamless adaptive robotic assistance according to the active contribution and movement ability of subjects. Different from existing compliance control of the rehabilitation devices, in this paper, it is achieved by directly influencing the nominal pressure of each actuator which will change inherent compliance of the soft ankle robot itself. Recent studies also claimed that the rehabilitation robot should keep a changeable assistance level to realize assistance-as-needed training for patient to maximum the recovery (Awad et al., 2017). There are plenty of fundamental studies on clinical rehabilitation support the idea of adaptive control, which means the robot assistance should be varied based on subject’s ability (Zariffa et al., 2012), or optimize the device characteristics on the basis of measured human performance (Zhang et al., 2017c). Several rehabilitation robot control strategies, namely, impedance (Fleerkotte et al., 2014; Jamwal et al., 2016), assistance-as-needed (Shahbazi et al., 2016; Hussain et al., 2017), patient cooperative (Duschau-Wicke et al., 2010b; Zhang et al., 2017b), and performance-based adaptive and game-evoked training (Burdea et al., 2013; Leconte and Ronsse, 2016) approaches have been developed in order to encourage more active contributions from the patient. Here, keeping this in mind, in addition to the compliance control in joint space, the admittance controller in task space is learning online the subject’s active effort and movement ability to adjust the robot compliance behavior, both approaches are combined to maximize the voluntary efforts. Furthermore, the variation of impedance control parameters and robot stiffness performance will determine the level of assistance that can be provided by the robot. The adaptive hierarchical compliance control scheme presents advantages compared to that with only joint space compliance or task space compliance based on a fact that the robot should be adjustably compliant in both end effector and joint space to meet different patients’ needs. For instance, adapting robot compliance according to patient active efforts and movement performance is able to make the rehabilitation process more comfortable and appropriate. This realizes the remarks of Reinkensmeyer and Dietz (2016) who suggested that robot-assisted rehabilitation with real-time adaptation and is challenged in a moderate but engaging way will likely help the patient receive appropriate training and better recovery.

Back to the experiments in Sections “Passive Control with Varying Robotic Compliance” and “Game Guided Adjustable Admittance Control,” which demonstrate the human–robot system controlled with only joint space compliance or task space compliance and results shown in Figures 11 and 12, we can see that the joint compliance adaptation or variable task admittance control strategy both have shown great potentials for compliant interactions. However, only joint space compliance control cannot generate encouraging training tasks, which means a changeable movement that can encourage patient’s exercise motivation is lacking, although the patient may receive different assistance levels from the robot. Similarly, without consideration of actuator’s inherent compliance property, the task space compliance control only has no difference from those implemented in rigid robots. In this situation, the robot stiffness performance and assistance output will keep unchanged. But for optimal human—robot interaction the robot assistance level should be adjusted by taking into account the actuator its own intrinsic compliance property (Cao et al., 2017). The proposed techniques in this study contribute to enhanced effectiveness of robot-assisted compliant rehabilitation by integrating joint compliance adaptation and task space admittance control. It adaptively modifies the robot assistance level based on real-time active torque measurement and meanwhile adjust the robot interaction admittance parameter based on patient’s movement ability to ensure training be encouraging and totally compliant. This can be verified by the experiment in Section “Adaptive Hierarchical Compliance Control” and the results shown in Figures 12 and 13 and Table 1. Note that all subjects’ movement errors including the patient’s are kept at a lower level during the late game sections and the levels of robot assistance are also decreased due to the reduction of nominal pressure and increase of robot admittance. It indicates that the proposed adaptive compliance control scheme adapts the robotic assistance both in joint space and task space according to the voluntary participation and movement performance of human subjects. To avoid the influence of human subject’s familiarity on the game training scores, each participant has been trained several times before the presented experiments. From phases A to D, when a healthy subject was involved, he was instructed to stimulate a patient recovered from highly impaired to healthy. A better result implies the better ability in generating more precise torque and movement. We also include the results of another two healthy participants, both show a similar pattern. Game training results of the patient show a slight improvement due the short-term training, which also indicates familiarity only cannot guarantee a good task performance, a good ability to control the foot with certain orientation and torque matters. Statistical analyses of the experimental results in Table 1 demonstrate that an increased active participation and movement ability would result in a reduced robotic assistance, and *vice versa*. Specifically, if a small level of active effort was measured from the human subject by using the end effector torque sensor, the controller would adapt the nominal pressure to an increased level so the compliance level is low and if a small movement deviation error is obtained, the controller will increase the robot admittance to allow more patient freedoms.

All subjects reported a kinematic constraint during the inactive game mode as the robotic compliance is set low and during the active mode, more kinematic freedom is found as the robot has a lower pressure and higher admittance. As a part of our previous work, Meng et al. proposed a robust iterative feedback tuning (IFT) controller for the CARR, to achieve a better and better trajectory tracking performance during the robot repetitive control (Meng et al., 2017a), while this control law was only for the movement control of the robot without human active interaction. A preliminary assessment of the adaptive compliance control scheme (without actuator compliance control) has also been carried out on a soft gait orthosis driven by PMAs (Hussain et al., 2013c). Later, Zhang et al. (2017b) presented an adaptive patient-cooperative control strategy on the CARR; however, the admittance adaptation scheme here was only implemented in the task space, just like most of current work on rigid robot compliance control. The robot was controlled to deviate from the reference trajectory when human–robot interaction exists, so it can be regarded as a trajectory adaptation scheme by considering human active effort. In other words, this study did not take the actuator inherent compliance property into account, also no consideration on the robot compliance response to patient’s behavior. Other recent work on soft rehabilitation robots (Jamwal et al., 2016; Hussain et al., 2017) also applied similar impedance control in only task space without considering the actuator’s variable compliance driving ability. Therefore, it is highly desirable to further explore the compliance property of the rehabilitation robots driven by soft actuators. One promising approach that has been widely accepted in rehabilitation robotics is to assist the patients only when they cannot complete the task by their own efforts, which means the robot should modify the amount of assistance according to the patient’s real-time performance (Zhang et al., 2017c). In this paper, we investigated two-level adaptive control strategies that aim to promote the subject’s participation during the robotic therapy. This compliance strategy modified the robot assistance output based on real-time estimation of subject’s active torque and movement ability. The active torque was obtained by the removal of estimated passive torque from measured torque and the movement ability was assessed on an error-based estimation. These methods were used to calculate the necessary robot assistance, resulting in adaptive compliance control where robot stiffness was adjusted automatically. A set of visual games was used to evaluate the control scheme’s performance. A key aspect of the control scheme lies in its two-level compliance adaptation, the estimate of the subject’s active torque and movement deviation as a measure of subject’s instantaneous ability. The strategy is able to instantly determine the amount of assistance needed by the subject from the position error value and the amount of active torque patient is currently providing. Unlike previous adaptive strategies, this scheme changes the robot assistance by modifying the compliance in both joint and task spaces for each instant of time (Koopman et al., 2013; Leconte and Ronsse, 2016). The proposed control scheme presents an advancement on the current compliance control schemes of the ARBOTs, especially those driven by compliant actuators. Experimental results obtained on a number of healthy and impaired subjects validated the effectiveness of the adaptive pressure-regulated and admittance-based compliance controller. Compared with the existing studies and our previous work, original contributions of this paper include the following aspects. One is a two-level hierarchical compliance controller that consists of not only an admittance controller in task space but also a compliance regulator in joint space. Next, a new method is proposed to build the relationship between soft actuators and their driven robot’s compliance performance. Third, we explored the adaptation control laws of the robot compliance and assistance level responding to patient’s active effort and movement ability in a game-guided training. Last but not least, we conducted pilot studies of the soft ARBOT controlled by the proposed training strategy on a number of healthy and impaired human subjects. These contributions and experimental findings suggest the great potential of such a new hierarchical compliance adaptation strategy as an efficient control solution for the soft robots in dynamic rehabilitation environment. To the best of the authors’ knowledge, the hierarchical compliance adaptation control on a soft rehabilitation robot with compliant muscle actuators has not been reported in the literature.

The results in this paper also show some limitations. The concept of estimated active torque of the subject is calculated by removing estimated passive torque from the total measurement. Pérez-Ibarra et al. (2015) first estimated the dynamic contribution of patient and then presented an adaptive impedance control strategy to realized assist-as-needed ankle rehabilitation. Similarly, though the model used in this work does not reveal the completely accurate ankle biomechanical features, the quantitative relationships observed between the estimated active torque and some parameters, verify that the movement ability relevant information of the patient can be estimated. Another limitation of this work is its small number of subjects involved. This study is a short-term trial that only try to verify the controller’s ability to stimulate patient’s voluntary efforts. However, this paper can be regarded as a preliminary step in testing the new hierarchical compliance control method for soft robots and can provide basic information for use in more complete studies. To evaluate the efficiency of the proposed strategy in practice, we are conducting experiments on a larger number of patients (Zhu et al., 2016) and will report the controller’s long-term effectiveness in the near future. On the other hand, to improve the analysis of the admittance performance, the human ankle’s model must include more higher order damping and inertia terms and biological components.

## Conclusion

In this paper, we proposed and evaluated a new hierarchical compliance control strategy for the soft ARBOT actuated by PMAs. A two-level compliance control was implemented by regulating the robot nominal pressure and admittance parameter based on the online estimation of subject’s active ankle torque and movement performance. Experiments were conducted on both healthy and impaired subjects, and results show that the proposed compliance control strategy can adjust the robot assistance level and motivate the subject’s active contribution to complete the tasks. The adaptive control scheme learnt the subject’s active effort and movement ability in real time and adapted the robotic compliance based on the estimation of human motor capacity. Many robots use impedance-based controllers to guide the subjects along given or modified trajectories. In this paper, a different strategy was implemented to provide variable robotic assistance at two compliance levels, a paradigm intended to fully explore the soft robots’ interaction capacity. There has not been previous work that implemented both joint space and task space compliance control schemes on PMA-driven ARBOT. In the future, rigorous clinical trials with more impaired subjects are necessary in order to validate therapeutic efficacy of the adaptive hierarchical compliance control scheme. This work also provides reference in further developing compliance control schemes for robots powered by soft actuators.

## Ethics Statement

This trial has been approved by the Human Participants Ethics Committees from Wuhan University of Technology, China and University of Auckland, New Zealand, and written informed consent was obtained from each participant.

## Author Contributions

QL and AL participated in the design of the study and the data collection, data analysis, and drafted the manuscript. WM participated in the design of the method, and participated as corresponding in the idea selection and helped in the data extraction and revising the manuscript. QA and SX participated as a supervisor to solve the discrepancies between AL and WM and to modify the manuscript. All authors read and approved the final manuscript.

## Conflict of Interest Statement

The authors declare that the research was conducted in the absence of any commercial or financial relationships that could be construed as a potential conflict of interest.
